# Characterization of Fatty Acid Metabolism in Lung Adenocarcinoma

**DOI:** 10.3389/fgene.2022.905508

**Published:** 2022-07-14

**Authors:** Suyu Wang, Aona Chen, Wanli Zhu, Di Feng, Juan Wei, Quanfu Li, Xuan Shi, Xin Lv, Meiyun Liu

**Affiliations:** ^1^ Department of Anesthesiology, Shanghai Pulmonary Hospital, Tongji University School of Medicine, Shanghai, China; ^2^ Department of Cardiothoracic Surgery, Changzheng Hospital, Naval Medical University, Shanghai, China; ^3^ Department of General Surgery, Changzheng Hospital, Naval Medical University, Shanghai, China; ^4^ Department of General Surgery, Shanghai Pulmonary Hospital, Tongji University School of Medicine, Shanghai, China

**Keywords:** fatty acid metabolism, lung adenocarcinoma, prognosis, fatty acid-related risk score, immunotherapy

## Abstract

**Background:** Lung adenocarcinoma (LUAD) is the most common subtype of non-small cell lung cancer. Fatty acid metabolism takes part in malignancy progression. However, the roles fatty acid metabolism plays in LUAD are still unclear.

**Methods:** The transcriptomic and clinical data of LUAD patients from The Cancer Genome Atlas (TCGA) and Gene Expression Omnibus (GEO) databases were extracted. ssGSEA, WGCNA, univariable Cox regression, and LASSO Cox regression analyses were performed to identify the fatty acid metabolism-related genes which influenced the overall survival (OS) and build a fatty acid-related risk score (FARS) model. A nomogram was established based on the FARS and other clinicopathological features, and ROC and calibration plots were used to validate the prediction accuracy. The tumor microenvironment (TME) of patients with high and low FARS was compared.

**Results:** A total of 38 genes were identified to be independently related to the survival outcome and put into a FARS model. High FARS patients exhibited significantly worse OS. The nomogram included the FARS and pathological stage, and the AUC of the nomogram predicting 1-, 2-, 3-, 4-, and 5-year OS was 0.789, 0.807, 0.798, 0.809, and 0.753, respectively. Calibration plots also indicated good accuracy. Moreover, the samples of the high FARS had higher expression of PDL1.

**Conclusion:** We constructed a FARS model which could accurately predict the survival outcome of the LUAD patients. The genes of the FARS are related to the tumor microenvironment and patients with high FARS can potentially benefit more from anti-PD1/PDL1 immunotherapy. In addition, the mechanisms of the genes in the FARS affecting prognosis are worthy of further research to develop new gene-targeted drugs.

## Introduction

Non-small cell lung cancer (NSCLC), the leading reason for cancer-related deaths, constitutes approximately 85% of malignant lung tumors (Bray et al., 2018). Lung adenocarcinoma (LUAD) is the most common subtype which accounts for nearly half of NSCLC (Chen et al., 2014). Moreover, LUAD is well-known for its heterogeneity in clinical, behavioral, cellular, and molecular features. Although most lung cancers are characterized by aggressive nature, almost 18.5% of lung cancers found by computed tomography (CT) screening are dormant and can lead patients to the hazard of overdiagnosis and overtreatment (Patz et al., 2014). Although significant effort has been made, the underlying cellular and molecular mechanisms of tumor behavior are still unclear, and long-term survival rates of lung cancer patients have been scarcely improved compared with other cancers (Siegel et al., 2016). Thus, it is important to detect new potential molecular signatures and therapeutic targets for LUAD.

Gene markers, particularly in tumor tissues, are dependable factors for predicting the long-term survival of cancer patients (Li et al., 2017; Hu et al., 2021). Therefore, detecting the molecular characterization which may cause poor outcomes can guide clinical adjuvant therapeutic strategies for a subgroup of patients who are at high risk. Moreover, this can be helpful in identifying new molecular targets for developing new medicines. The public database of gene expression of large cohorts of patients facilitates the aim of establishing a metabolic gene signature for predicting survival outcomes and analyzing the tumor microenvironments (TMEs).

Attributed to the fast proliferation of cancer cells and insufficient angiogenesis, the main characteristics of TMEs are malnutrition, hypoxia, high oxidation, and acidity. Hence, compared to normal cells, tumor cells manifest distinct metabolic features to cope with diverse deleterious microenvironments *via* metabolic recoding processes which maintain the proliferation and survival of tumor cells when the oncogenic signal is blocked (Lue et al., 2017). Reprogramming of energy metabolism, known as a hallmark of cancers, has been lately verified to take part in the initiation, progression, and drug resistance in lung cancer (Hensley et al., 2016; Chen et al., 2019). There is a distinct difference in carbohydrate, amino acid, and lipid metabolism between tumor cells and normal cells (Yu et al., 2019). Taking carbohydrate metabolism as an example, normal tissue cells decompose glucose into pyruvate by glycolysis, and in addition to glucose decomposition, oxidative phosphorylation in mitochondria generates vast energy. On the other hand, in cancer cells, the glucose is catabolized into lactate with an insufficient generation of energy; thus, cancer cells consume much more glucose than normal cells (Faubert et al., 2017). As demonstrated by Xue et al., cellular pyruvate metabolism of LUAD changed, including the reduction of expression of mitochondrial pyruvate carrier 1 (MPC1) compared with adjacent normal tissues. Xue et al. (2021) also revealed that higher MPC1 expression was related to a favorable prognosis. In addition to carbohydrate metabolism, lipid metabolism is also a potential hallmark for cancers. Lipogenesis, lipid uptake, and lipid storage are highly upregulated in malignant tumors to meet the augmented demands of membrane biogenesis and promote cancer cell proliferation and survival, especially under conditions of insufficient nutrition and oxygen (Menendez and Lupu, 2007; Nath et al., 2015; Qiu et al., 2015; Röhrig and Schulze, 2016; Geng and Guo, 2017). Presently, fatty acid metabolism, involved in many biological activities including signaling molecule synthesis, cell membrane formation, and energy storage in carcinogenesis, has been widely researched (Currie et al., 2013). For example, Ding et al. (2021) exhibited that the signature of fatty acid metabolism can predict the prognosis of colorectal cancer and was associated with chemoresistance and TME characteristics. In another study conducted by Svensson et al. (2016), ND-646, an allosteric inhibitor of the acetyl-CoA carboxylase (ACC) enzymes ACC1 and ACC2 which suppress ACC subunit dimerization, prevented the synthesis of fatty acid *in vitro* and *in vivo*. Thus, ND-646 significantly inhibited lung cancer growth in the KRAS p53 and KRAS Lkb1 mouse models of NSCLC, indicating the therapeutic potential of the ACC inhibitor in malignant tumors (Svensson et al., 2016). Based on previous studies, it is clear that an analysis of the metabolic pathway of lung cancer can help us comprehend the molecular mechanism of lung cancer and develop novel personalized therapeutic regimens (Sayin et al., 2019). However, the characterization of the genes related to fatty acid metabolism in LUAD has not been systematically investigated.

To detect the underlying genomic mechanism of fatty acid metabolism of LUAD, we used the genomic information on the clinicopathological features of 1,087 LUAD patients from The Cancer Genome Atlas (TCGA) and Gene Expression Omnibus (GEO) database to reveal the pattern of fatty acid metabolism and establish a fatty acid-related risk score (FARS) model. The FARS model proved to be an independent prognostic factor in the survival outcome of LUAD patients. Moreover, the FARS can recognize patients who are suitable for anti-PD1/PDL1 antibody immunotherapy, indicating that fatty acid metabolism is highly related to individual characterizations of the TME. All these findings give a new insight into lipid metabolic mechanism and potential therapeutic targets.

## Material and Methods

### Data Acquisition and Processing

The clinicopathological features and fragments per kilobase per million mapped reads (FPKM) of 573 LUAD samples were extracted from UCSC Xena (http://xena.ucsc.edu/; accessed October 8 2021). The exclusion criteria were as follows: patients (a) with no survival information or survival time less than 30 days; (b) with no age, sex, or the American Joint Committee on Cancer Tumor Node Metastasis (AJCC TNM) stage information; and (c) has received neoadjuvant therapy. As a result, 468 LUAD samples with complete clinicopathological characteristics, including age, sex, AJCC TNM stage, and overall survival (OS) data, were put into the analysis as the training cohort.

The microarray dataset GSE72094 was downloaded from GEO datasets (https://www.ncbi.nlm.nih.gov/gds/) and was used as the first validation cohort. This dataset was produced by using a Rosetta/Merck Human RSTA Custom Affymetrix 2.0 microarray and contained 393 samples of lung adenocarcinoma. In addition, 226 lung adenocarcinoma samples from GSE31210 [(HG-U133_Plus_2) Affymetrix Human Genome U133 Plus 2.0 Array] were used as the second independent validation cohort. The microarray and RNA-seq data in this study were normalized and log_2_ transformed.

### Fatty Acid-Related Pathways and Biological Processes

A total of 68 fatty acid-related pathways or biological processes were extracted from hallmark gene sets (H collection), curated gene sets (C2 collection), and ontology gene sets (C5 collection) in Molecular Signatures Database (MSigDB, http://software.broadinstitute.org/gsea/index.jsp). All these pathways, biological processes, and their corresponding genes were fused into a file in CSV format (Supplementary Material S1). The related infiltration and activity levels of those pathways or biological processes in LUAD samples were quantified using the single-sample Gene Set Enrichment Analysis (ssGSEA) in the R package GSVA and normalized by using the Z-score method. The univariate and multivariate Cox analyses were used to assess the significance of these pathways or biological processes, and a two-side *p*-value <0.05 was considered to be statistically significant.

### Candidate Gene Selection and Signature Construction in the Training Cohort

Using the R package WGCNA, weighted gene co-expression network analysis (WGCNA) was performed. The analysis was performed on the top 5,000 genes with the highest standard deviation by the standard protocol. A gene correlation matrix with an optimal soft thresholding of power was used to derive the adjacency matrix. Modules were obtained with the following criteria: module size ≥30 and height for merging modules ≥0.2. The relationship between the modules and fatty acid-related pathways or biological processes was calculated based on ssGSEA scores, and modules correlated with fatty acid-related pathways or biological processes were extracted based on *p*-value < 0.05 and | r | > 0.3.

Thereafter, the genes related to prognosis in those modules were selected by the univariate Cox analysis. Then, the least absolute shrinkage and selection operator (LASSO) regression model was applied to further detect the most robust prognostic markers. A fatty acid-related risk score (FARS) of each sample was established using the formula

$$FARS=\overset{1}{\underset{i}{\sum }}Coefficient\left(mRNAi\right)\times \;Expression\left(mRNAi\right).$$

### Predictive Power of the Fatty Acid-Related Risk Score in Training and Validation Cohorts

A violin plot was drawn to compare the FARS of the dead and alive patients in the training cohort. Then, the FARS was divided into high-score and low-score groups using the median value as the cut-off value. For different risk score groups in the training cohort, the Kaplan–Meier curve, risk factor curve, and survival status scatter plot were plotted. To authenticate the predictive power of the FARS in the training cohort, the area under the curve (AUC) according to 3- and 5-year OS was calculated and plotted, respectively, using the survivalROC package. In addition, the predictive performance and applicability of the FARS were further verified in the two validation cohorts.

### Subgroup Analysis of the Fatty Acid-Related Risk Score in Different Clinicopathological Features

To determine the robustness of the FARS for predicting the survival outcome in different subgroups of clinicopathological features containing age, sex, and pathological stage (pStage), the Kaplan–Meier curve was plotted to evaluate the discriminative capacity of the FARS based on the median value.

### Immune Profile

To explore the relationship between immune status and the FARS, the expression of 22 immune cells was calculated in high and low FARS groups using the CIBERSORT package. Furthermore, the expression levels of the immune checkpoint containing PD-1, PD-L1, and CTLA4 of high and low FARS groups were compared.

### Establishment of a Prognostic Nomogram

All samples of the training cohort from TCGA database were used to construct the nomogram. The FARS and clinicopathological features were included in the univariate and multivariate Cox analyses with *p*-value <0.05 as the screening criterion. The variables selected were included in the nomogram using the “RMS” package; the predictive performance of the nomogram was assessed by the time-dependent receiver operating characteristic curves (tROC) and calibration curves.

### Statistical Analysis

R software (www.r-project.org) was used in statistical analyses. Manuals for the R packages used in the present study could be downloaded from the Internet (https://cran.r-project.org/web/packages/available_packages_by_name.html). Clinicopathological characteristics of the training and validation cohorts were compared using the Kruskal–Wallis test and chi-square test as appropriate.

## Results

### Data Processing

Expression data of FPKM and clinicopathological features of 468 LUAD cancer samples from TCGA were selected and downloaded according to the screening criteria, and these samples were analyzed as the training cohort. A total of 393 cancer samples in GSE72094 were the first validation cohort and the corresponding expression data and clinicopathological features were downloaded using the GEOquery package. Similarly, the information of 226 samples from GSE31210 was obtained as the second validation set. The clinicopathological characteristics of these three cohorts are presented in Supplementary Material S2. A total of 170 (36.3%), 111 (28.2%), and 35 (15.5%) patients died in the training, first validation, and second validation cohorts, respectively. The median (interquartile range) follow-up times for these three cohorts were 658 (435-1118), 825 (541-1012), and 1744 (1246-2050) days, respectively.

### Identification of Prognosis-Related Pathways and Biological Processes

**FIGURE 1:**
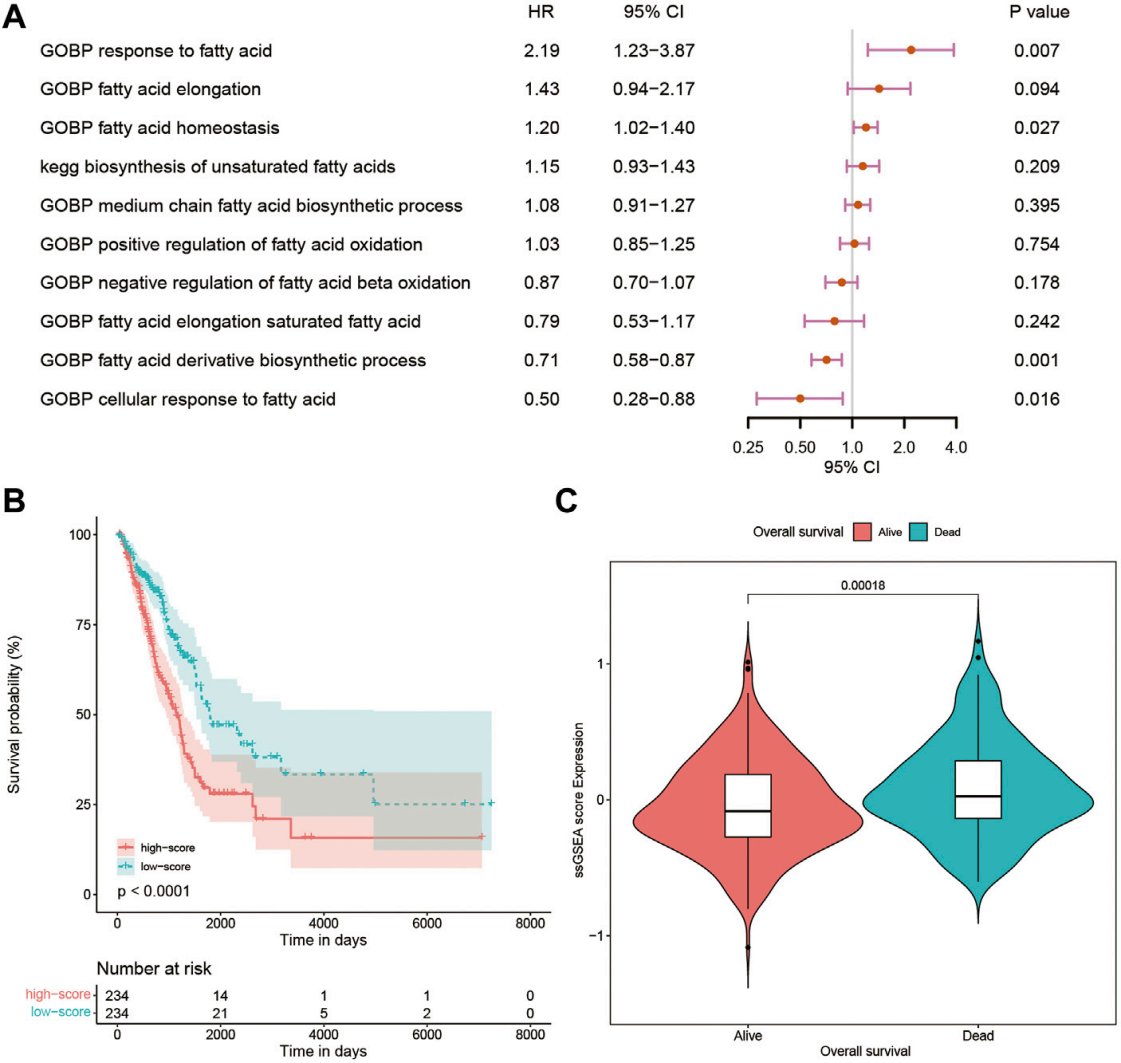
**(A)** Multivariate Cox analysis of 10 pathways and biological processes; **(B)** Kaplan–Meier curves of patients with high and low ssGSEA scores; **(C)** violin plot comparing ssGSEA scores of survival and dead patients. GOBP, gene ontology biological process; ssGSEA, single-sample Gene Set Enrichment Analysis.

Using the CSV file of fatty acid-related pathways and biological processes and the method of ssGSEA, separate enrichment scores for each sample from LUAD were calculated. A total of 10 pathways and biological processes were selected for the univariate Cox analysis in terms of *p*-value < 0.05. Finally, only four biological processes, namely, gene ontology biological process (GOBP) response to fatty acid, GOBP fatty acid homeostasis, GOBP fatty acid derivative biosynthetic process, and GOBP cellular response to fatty acid proved to be independent risk factors in the multivariate Cox analysis (Figure 1A). The Kaplan–Meier curve and violin plot demonstrated similar results that the ssGSEA score was related to prognosis (Figures 1B,C).

### Establishment of the Fatty Acid-Related Risk Score

**FIGURE 2:**
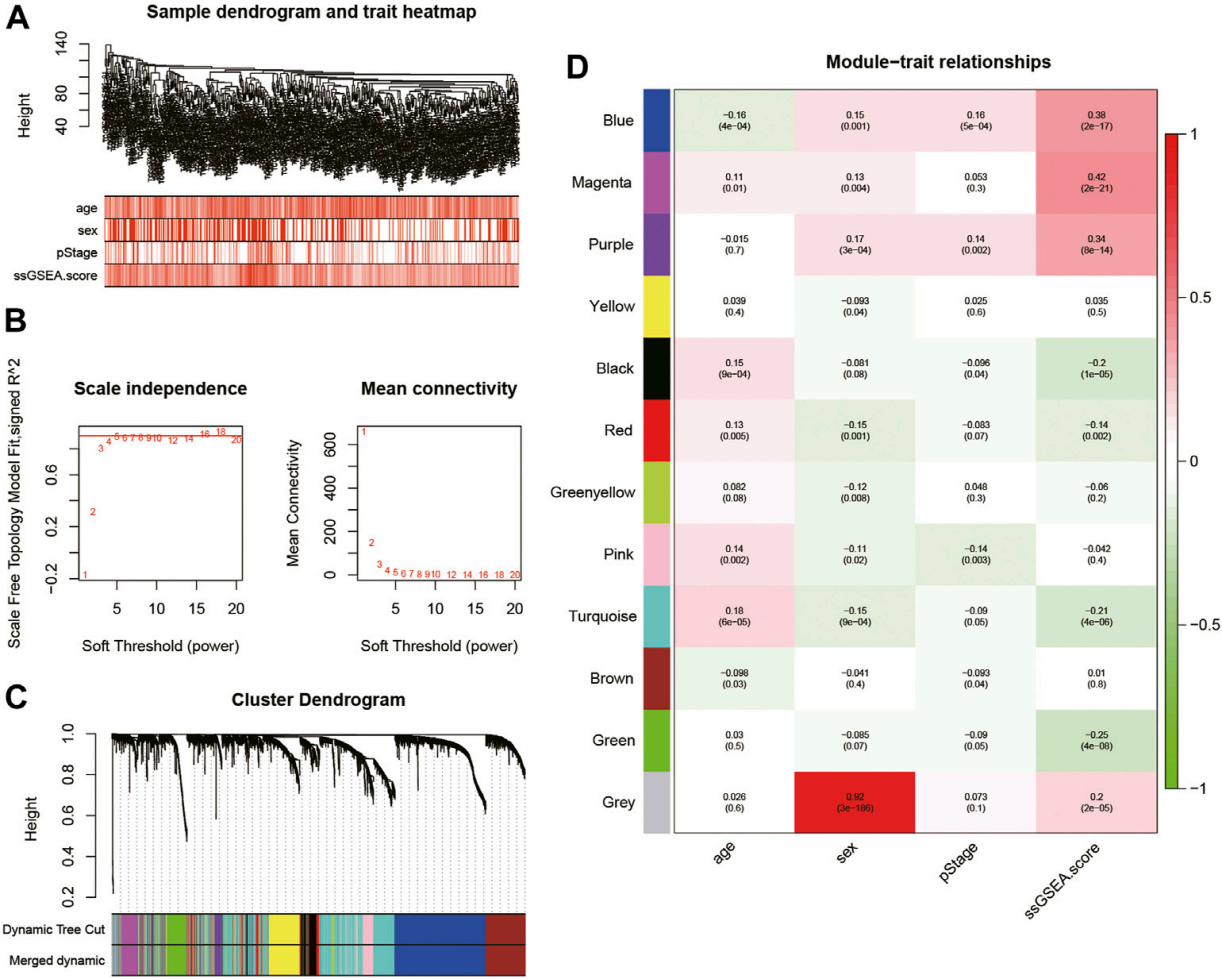
**(A)** Cluster was based on the expression data of the training cohort, which contained 468 lung adenocarcinoma samples. The top 5,000 genes with the highest standard deviation were used for the analysis by WGCNA. The color intensity was proportional to age, sex, pStage, and ssGSEA score; **(B)** detection of the optimal soft thresholding power; **(C)** cluster dendrogram of genes in the training cohort. Every branch in the figure denotes one gene and each color below denotes one co-expression module; **(D)** heatmap of the relationship between module eigengenes and the ssGSEA score. The blue, magenta, and purple modules were most positively correlated with the ssGSEA score. WGCNA, Weighted Gene Co-expression Network Analysis; ssGSEA, single-sample Gene Set Enrichment Analysis.

WGCNA was performed on the top 5,000 genes with the highest standard deviation in TCGA cohort to explore the genes correlated with the fatty acid-associated pathways, and the sample dendrogram was exhibited (Figure 2A). The quality of all samples was very good. The optimal soft thresholding power of 4 was selected to obtain the adjacency matrix (Figure 2B). A total of 12 modules were constructed under the minimum module size of 30 and there was no module merged with the minimum height for merging modules of 0.2 (Figure 2C). In Figure 2D, the blue, magenta and purple modules met the criteria of *p*-value <0.05 and | r | > 0.3.

**FIGURE 3:**
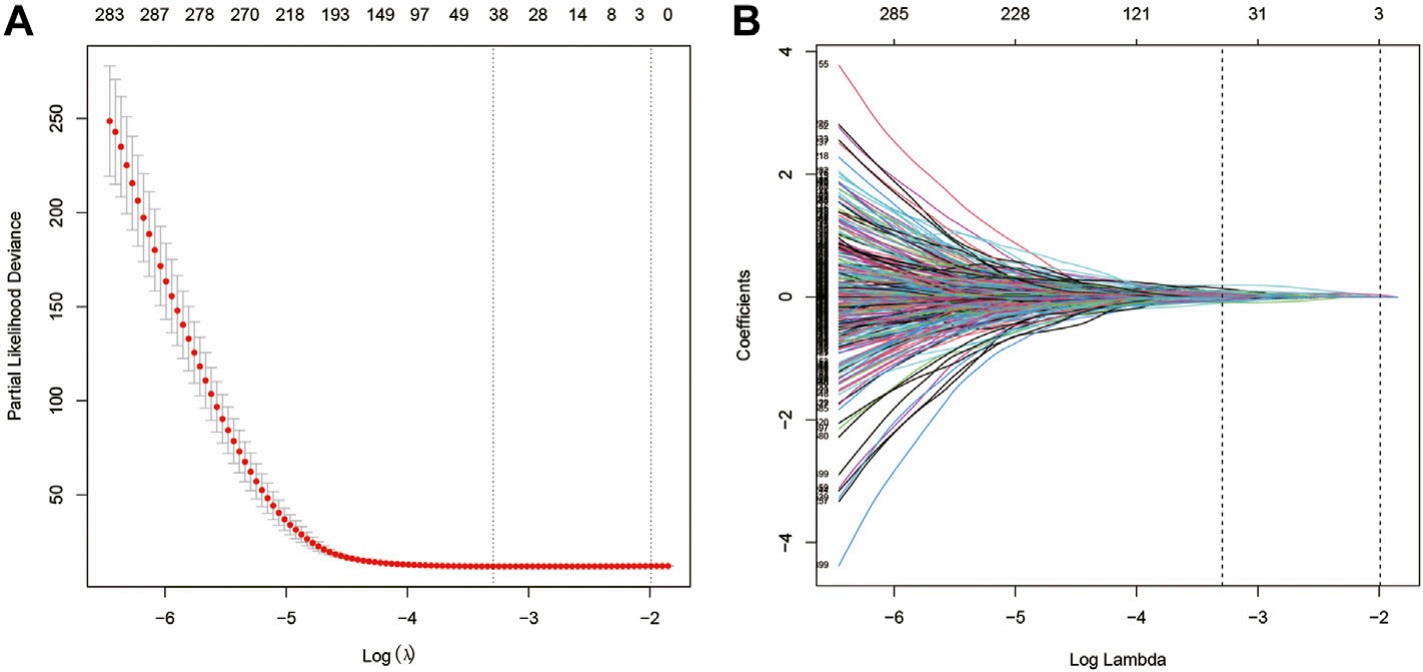
**(A)** Screening of optimal parameters (lambda) at which the vertical lines were drawn; **(B)** Lasso coefficient profiles of the fatty acid metabolism-related genes with non-zero coefficients determined by the optimal lambda.

Univariate Cox analysis was performed for each 1,567 genes in the three modules, and those 653 genes with *p*-value <0.05 in the univariate analysis were included in the LASSO regression analysis (Supplementary Material S3) to construct the FARS. The ten-fold cross-validation was used to determine the optimal penalty parameter (λ) of the model (Figure 3). A total of 38 genes (LDHA, TM4SF1, HPCAL1, P4HA1, TP53I3, HGSNAT, MYO6, SQLE, IVD, KLHDC8B, GNPNAT1, PAQR4, ENPP5, JAG1, MCTP2, PLEKHA6, MAOB, ANKRD29, ELOVL6, ABAT, ZNF738, BEX5, LETM2, WASF1, INPP5J, DKK1, SLC4A5, CDC25C, FAIM2, BAIAP2L2, GPR37, TM4SF4, TCN1, GALNT13, CNTNAP2, IGFBP1, IGF2BP1, and SALL1) were included in the LASSO model. The coefficient of each gene is given in Supplementary Material S4.

### Predictive Power of the Fatty Acid-Related Risk Score in Training and Validation Cohorts

**FIGURE 4:**
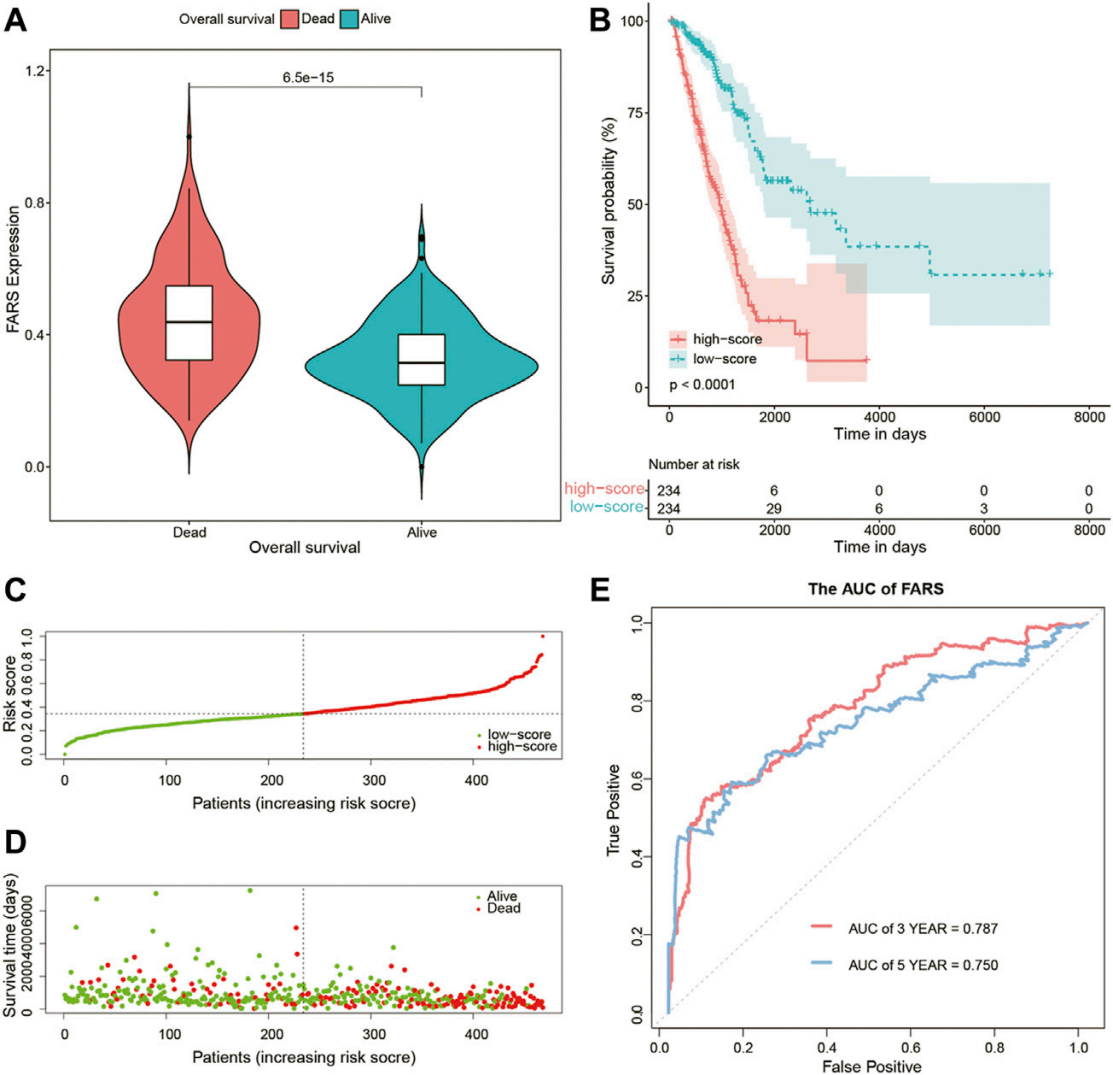
**(A)** Violin plot comparing the FARS of survival and dead patients for the training cohort; **(B)** Kaplan–Meier curves of patients with high and low FARS for the training cohort; **(C)** distribution patterns of risk scores for the training cohort; **(D)** distribution patterns of survival status for the training cohort; **(E)** ROC curves of the FARS for the training cohort. FARS, fatty acid-related risk score; ROC, receiver operating characteristic.

As shown in Figure 4A, the dead patients had a significantly higher FARS than the alive patients during the follow-up period. A total of 468 samples of the training cohort were divided into the high FARS group and low FARS group by the median value of the FARS, and the high FARS group exhibited worse OS than the low FARS group, with *p*-value <0.01 (Figure 4B). Moreover, the plot distribution patterns of risk scores and survival status showed good results (Figures 4C,D). As the FARS increased, the OS time decreased and mortality increased. Calculated by the “survivalROC” package, the 3- and 5-year AUC (AUC = 0.787 and 0.750) of the FARS is presented in Figure 4E.

**FIGURE 5:**
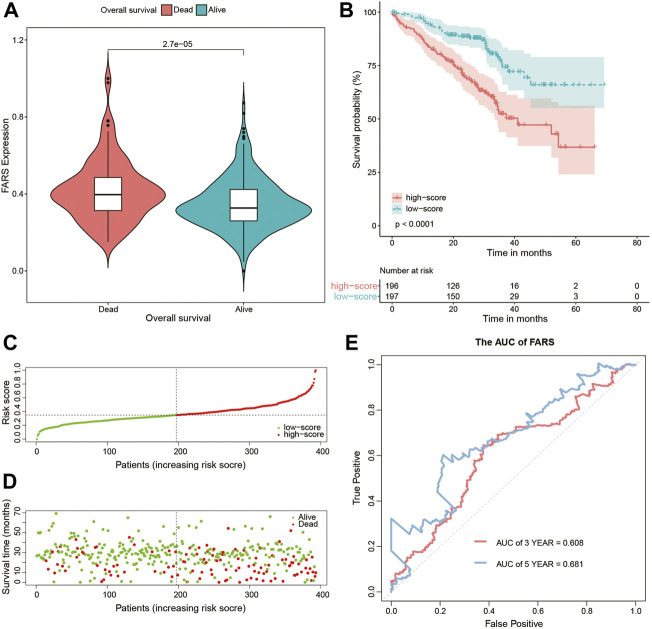
**(A)** Violin plot comparing the FARS of survival and dead patients for the first validation cohort (GSE72094); **(B)** Kaplan–Meier curve of patients with high and low FARS for the first validation cohort (GSE72094); **(C)** distribution patterns of risk scores for the first validation cohort (GSE72094); **(D)** distribution patterns of the survival status for the first validation cohort (GSE72094); **(E)** ROC curves of the FARS for the first validation cohort (GSE72094). FARS, fatty acid-related risk score; ROC, receiver operating characteristic.

**FIGURE 6:**
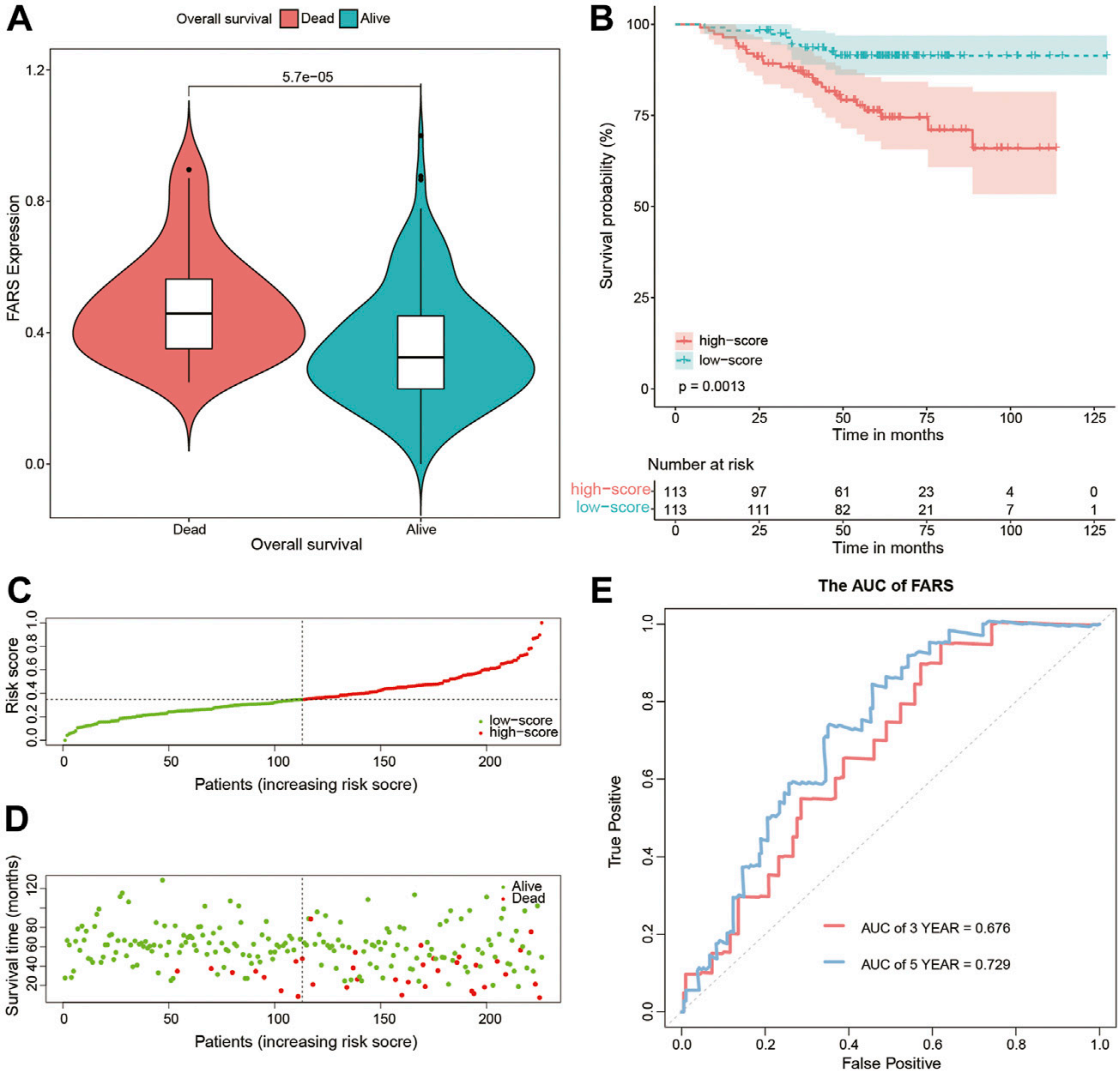
**(A)** Violin plot comparing the FARS of survival and dead patients for the second validation cohort (GSE31210); **(B)** Kaplan–Meier curves of patients with high and low FARS for the second validation cohort (GSE31210); **(C)** distribution patterns of risk scores for the second validation cohort (GSE31210); **(D)** distribution patterns of the survival status for the second validation cohort (GSE31210); **(E)** ROC curves of the FARS for the second validation cohort (GSE31210). FARS, fatty acid-related risk score; ROC, receiver operating characteristic.

These results indicated that the high FARS showed a worse prognosis, and FARS had a good performance in predicting the prognosis of LUAD patients. The same result was also demonstrated in the two validation cohorts. The FARS of the two validation cohorts was calculated according to the LASSO formula. The dead patients had significantly higher FARS than the alive patients in the two validation cohorts (*p*-value <0.01, Figure 5A, Figure 6A). The high FARS patients showed a worse OS compared with the low FARS patients (*p*-value <0.01, Figures 5B, 6B). The trend of the survival time and mortality in the validation cohorts was similar to those in the training cohort (Figures 5C,D, 6C,D). We also found that the 3- and 5-year AUC (AUC of validation 1 = 0.608, 0.681; AUC of validation 2 = 0.676, 0.729) of the FARS was similar to those of the training group (Figures 5E, 6E).

### Subgroup Analysis of the Fatty Acid-Related Risk Score in Different Clinicopathological Features

**FIGURE 7:**
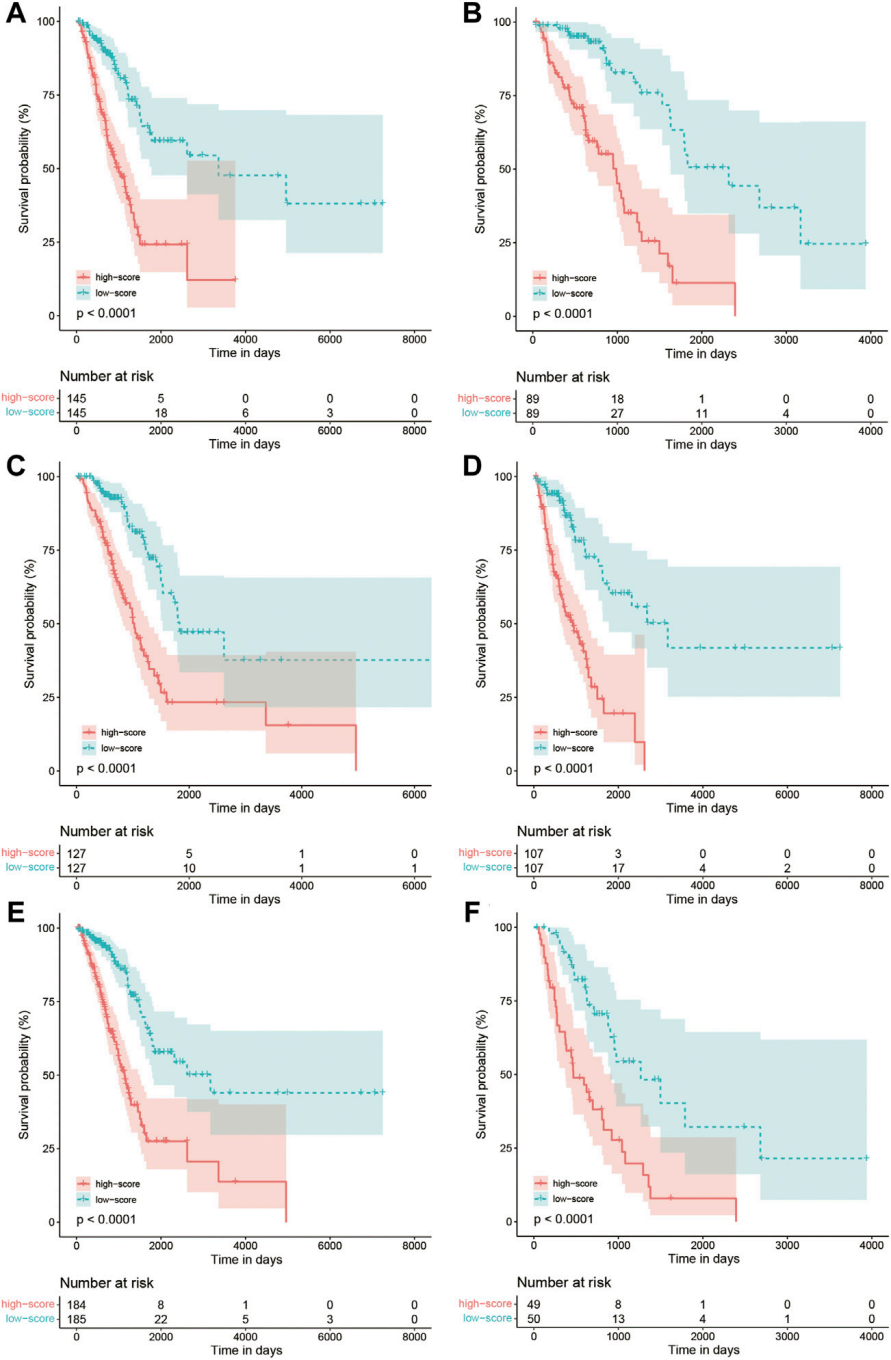
**(A)** Kaplan–Meier curves of patients with age<70 in the training cohort comparing high and low FARS; **(B)** Kaplan–Meier curves of patients with age ≥ 70 in the training cohort comparing high and low FARS; **(C)** Kaplan–Meier curves of female patients in the training cohort comparing high and low FARS; **(D)** Kaplan–Meier curves of male patients in the training cohort comparing high and low FARS; **(E)** Kaplan–Meier curves of I–II pStage patients in the training cohort comparing high and low FARS; **(F)** Kaplan–Meier curves of III–IV pStage patients in the training cohort comparing high and low FARS. FARS, fatty acid-related risk score; pStage, pathological stage.

The Kaplan–Meier curve comparing the survival outcome of high and low FARS in subgroups of clinicopathological features including age, sex, and pStage is shown in Figures 7A–F, where the high FARS patients exhibited worse OS than the low FARS patients in each subgroup.

### Immune Profile

**FIGURE 8:**
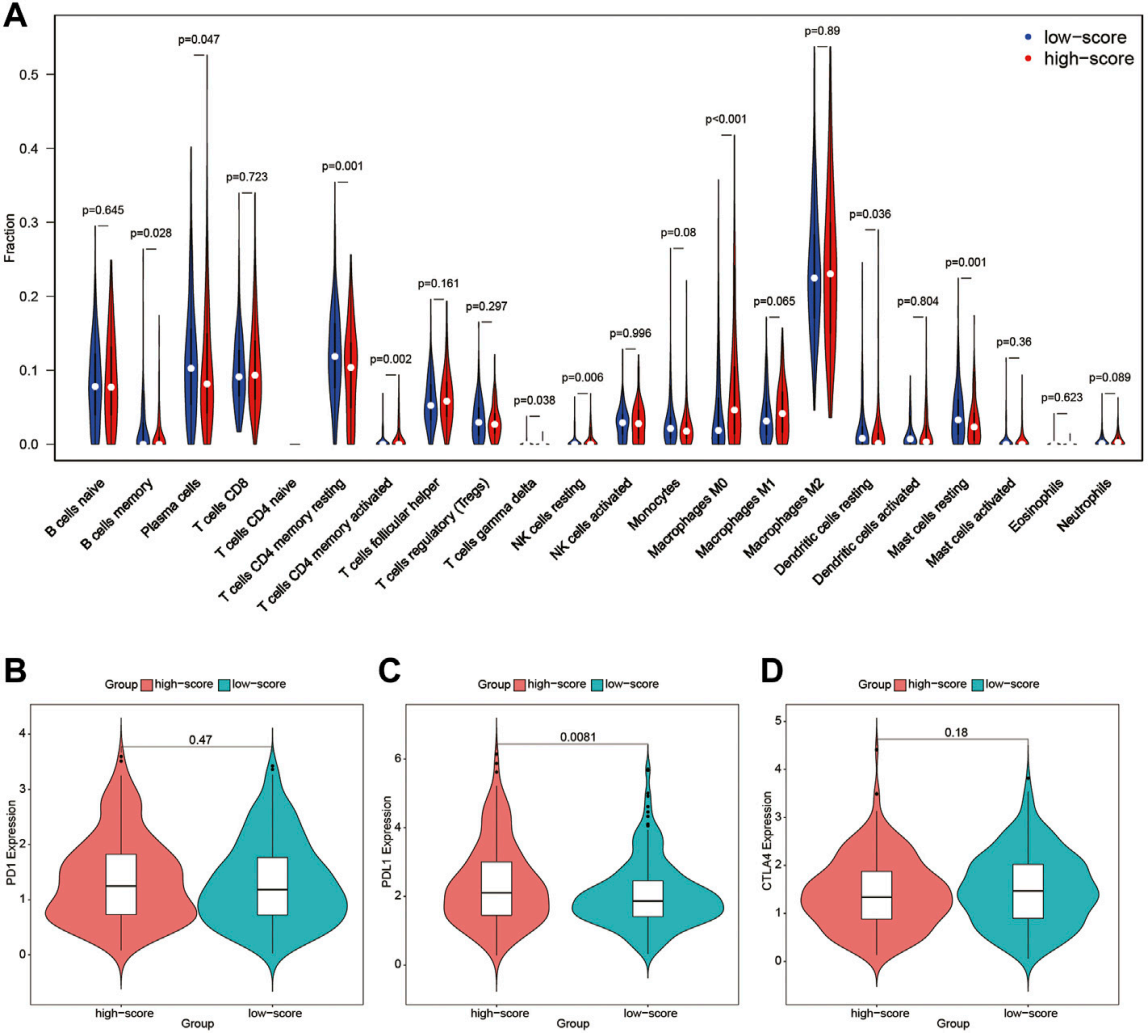
**(A)** Immune profile of high and low FARS; **(B)** expression of programmed cell death protein 1 (PD1) of high and low FARS; **(C)** expression of programmed death-ligand 1 (PDL1) of high and low FARS; **(D)** expression of cytotoxic T-lymphocyte-associated protein 4 (CTLA4) of high and low FARS. FARS, fatty acid-related risk score.

To further detect the correlation between the immune profile and FARS, the degree of immune cell infiltration of the high and low FARS groups was compared (Figure 8A). B cells memory, plasma cells, T cells CD4 memory resting, T cells gamma delta, dendritic cells resting, and mast cells resting were significantly more prevalent in the low FARS patients. Whereas, T cells CD4 memory activated, NK cells resting, and macrophages M0 were significantly more prevalent in the high FARS patients.

The expression of immune checkpoint inhibitors targeting immune checkpoint proteins including programmed cell death protein 1 (PD1), programmed death-ligand 1 (PDL1), and cytotoxic T-lymphocyte-associated protein 4 (CTLA4) was also calculated in high and low FARS patients (Figures 8B–D). The high FARS group exhibited higher expression of PDL1 (Figure 8C), suggesting that patients with a high FARS may be more susceptible to immune checkpoint inhibitors targeting PD1/PDL1.

### Establishment of a Prognostic Nomogram

**FIGURE 9:**
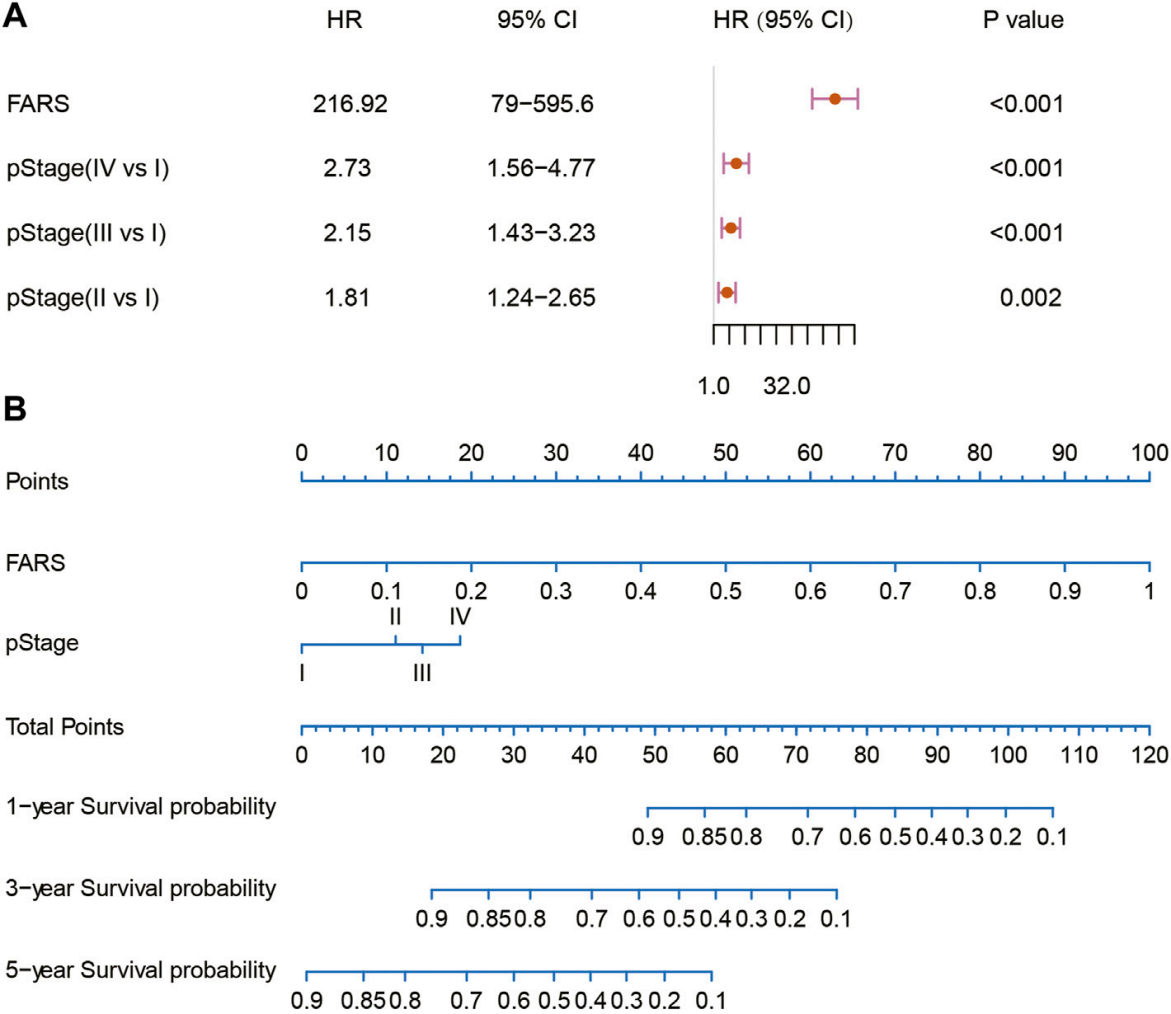
**(A)** Multivariate Cox analysis of the variables selected for the nomogram; **(B)** nomogram predicting 1-, 3-, and 5-year OS for LUAD patients. OS, overall survival; LUAD, lung adenocarcinoma.

**FIGURE 10:**
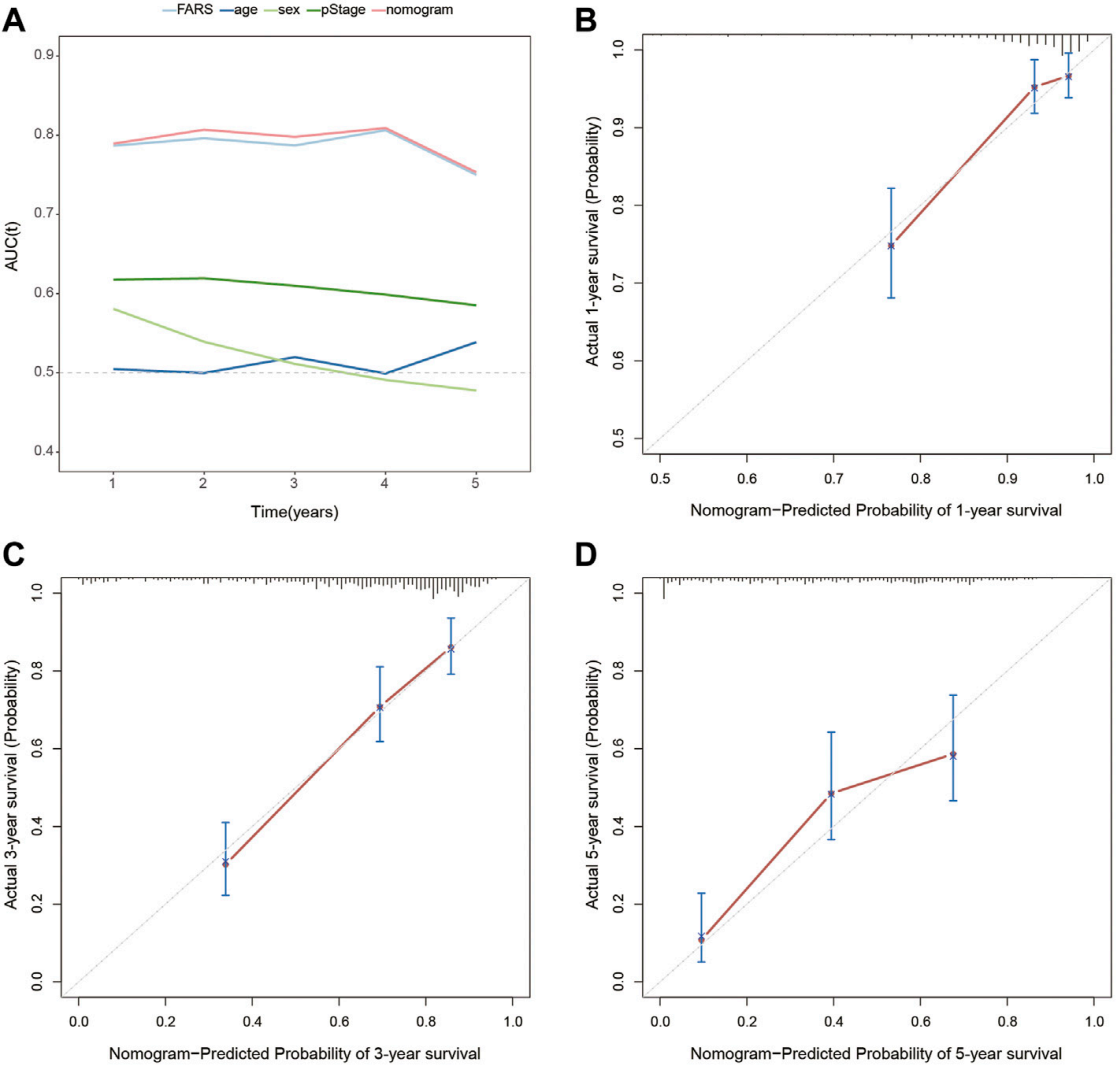
**(A)** Time-dependent AUC for the FARS, age, sex, pStage, and nomogram; **(B)** calibration plot for 1-year OS prediction of the nomogram; **(C)** calibration plot for 3-year OS prediction of the nomogram; **(D)** calibration plot for 5-year OS prediction of the nomogram. AUC, area under the curve; FARS, fatty acid-related risk score; pStage, pathological stage; OS, overall survival.

In the end, a nomogram was established to predict the OS more conveniently. The FARS and clinicopathological features of all TCGA samples were included in the univariate and multivariate Cox analyses, and FARS and pStage proved to be independent risk factors of LUAD (*p*-value <0.01). A nomogram based on the FARS and pStage was plotted using the “rms” package (Figures 9A,B). The tAUC of FARS, clinicopathological characteristics, and nomogram is shown in Figure 10A. The figure showed that the prediction ability of the FARS (AUC = 0.787, 0.796, 0.787, 0.806, and 0.750) was higher than that of other clinicopathological features and the predictive performance of the nomogram (AUC = 0.789, 0.807, 0.798, 0.809, and 0.753) was higher than that of other features. Furthermore, the calibration curves of the nomogram predicting 1-, 3-, and 5-year OS were plotted, and the predicted OS probability was very close to the actual OS probability in each calibration curve (Figures 10B–D).

## Discussion

Metabolic reprogramming, as a defining characteristic of malignant tumors, plays an important role in cancer development and opens up novel opportunities for cancer therapies (Kapadia et al., 2018). Lipid metabolism has been demonstrated as a pivotal regulator of malignant tumor immunology (Chen et al., 2021a). Most malignant tumor cells exhibit characteristic modifications in *de novo* lipid biosynthesis, lipogenic characteristics, and lipid metabolism (Wymann and Schneiter, 2008; Santos and Schulze, 2012). Upregulated lipid metabolism is one of the physiological features of human cancers and supports signal molecule synthesis and transduction and energy generation (Zaugg et al., 2011; Louie et al., 2013). Molecules regulating lipid metabolism can be potential therapeutic targets. For example, Montal et al. (2015) revealed that phosphoenolpyruvate carboxykinase (PEPCK) promotes lipid synthesis in cancer cells, helping to coordinate a pivotal feature of cancer metabolism. In addition, Svensson et al. (2016)’s study proved that the ACC inhibitor, ND-646, could prevent the biosynthesis of fatty acids in a mouse model of NSCLC. Blockage of fatty acid oxidation leads to the death of lung and ovarian cancer cells (Sullivan et al., 2014). Inhibition of fatty acid oxidation can enhance cancer therapies by modulating the immunosuppressive functions of myeloid-derived suppressor cells (Hossain et al., 2015). Previous studies focused on the influence of a single regulator of pathways related to fatty acid metabolism; however, the comprehensive roles of genes related to fatty acid metabolism have not been presented.

Until now, this is the first research to investigate the relationship between LUAD and fatty acid metabolism-related genes. Based on TCGA and GEO dataset and using the methods of ssGSEA, WGCNA, univariable Cox regression model, and LASSO Cox regression model, a FARS model with 38 fatty acid metabolism-related genes was built and proved to be an independent predictive factor for the survival outcome of LUAD patients. The high-risk score patients showed worse OS than low-risk score patients both in TCGA cohort and two GEO cohorts. Subgroup analyses of different clinicopathological features confirmed the stable prediction of the FARS model. These results indicated the FARS could discriminate patients with poor prognoses. Furthermore, compared to age, gender, and pStage, the FARS model had a larger AUC. As a matter of fact, the AUC of the FARS in predicting survival probability within 5 years was as high as close to 0.8, marking a relatively accurate prediction. Based on the FARS and pStage, a nomogram was established and the calibration plots exhibited good accuracy. It was interesting to find that the FARS model and the nomogram combining the pStage and FARS had a similar AUC, indicating that the pStage brought little improvement to the model. As shown in the nomogram, the FARS contributes much more than the pStage in the points, which means that the FARS maybe more important than the pStage in predicting the survival outcome of LUAD patients.

Of all these 38 genes included in the FARS, 30 have been reported to be related to LUAD/NSCLC in the PubMed database. However, no research studies were found to concentrate on the relationship between LUAD with the other eight genes: HGSNAT, ENPP5, MCTP2, PLEKHA6, ANKRD29, ZNF738, SLC4A5, and CNTNAP2. ENPP5, MCTP2, PLEKHA6, ANKRD29, SLC4A5, and CNTNAP2 were shown to play a role in malignancy other than NSCLC (Bralten et al., 2010; Langevin et al., 2012; Smith et al., 2012; Parris et al., 2014; Yang and Loh, 2019; Gopalakrishnan et al., 2020; Song et al., 2020; Sun et al., 2020; Chen et al., 2021b). No previous research studies were published concerning the relationship between cancer and HGSNAT or ZNF738. Overall, the underlying mechanisms of these genes included in the FARS relating to fatty acid metabolism and LUAD prognosis are worth further investigation.

The genes with the largest hazard ratio with a value >1 in the FARS were LDHA, HPCAL1, and IGF2BP1. MYO6, BEX5, and ABAT exhibited the smallest hazard ratio with a value <1. LDHA is a critical enzyme which can catalyze the mutual transformation of lactic acid and pyruvic acid in glycolysis (Massari et al., 2016), and pyruvic acid can be converted into acetyl-CoA which is the substrate for fat synthesis. As reported in previous studies, malignant tumors have a higher level of lipogenesis (Menendez and Lupu, 2007; Röhrig and Schulze, 2016). In the present study, higher expression of LDHA indicated worse OS, which suggests that higher LDHA in LUAD might help enhance fat synthesis by promoting acetyl-CoA generation in glycolysis, thus providing more energy for tumor progression. HPCAL1 was the gene with the second-largest hazard ratio in our study, and interestingly, a recent study demonstrated that HPCAL1 could directly bind to LDHA and enhance its activation, thus influencing fatty acid synthesis and promoting NSCLC growth (Wang et al., 2022). IGF2BP1 was reported to be associated with lipid accumulation in macrophages or serve as a biomarker for NSCLC; however, the relationship between IGF2BP1 and fatty acid metabolism in LUAD had not been shown (Kato et al., 2007; Liu et al., 2022). As for MYO6, its relationship with fatty acid metabolism or LUAD was also obscure. The expression of BEX5 was significantly decreased in several LUAD cell lines compared with normal lung epithelial cells *in vitro* and also downregulated in LUAD tissues compared with adjacent normal tissues (Zhang et al., 2019), while the association between BEX5 and fatty acid metabolism in LUAD was never studied. A previous study revealed that ABAT was related to the survival of LUAD patients, but no research disclosed whether ABAT influenced LUAD survival *via* fatty acid metabolism (Wang et al., 2021).

As immunotherapy has drastically improved the survival outcome of many cancer patients as compared to chemotherapy or radiotherapy, more studies concerning the relationship between fatty acid metabolism and immunotherapy were carried out recently (Bleve et al., 2021). For instance, in a Lewis lung carcinoma model, inhibiting CTP1, a rate-limiting enzyme in the fatty acid oxidation cycle, could significantly enhance adoptive cell transfer therapy and reduce tumor progression (Hossain et al., 2015). Another study revealed that lipofermata could reduce the uptake of fatty acid by polymorphonuclear myeloid-derived suppressor cells and suppress tumor progression when combined with anti-CTLA4 or anti-PD1 antibodies in lung carcinoma models (Veglia et al., 2019). Several other studies also proved that targeting fatty acid metabolism could inhibit the immunosuppressive function of myeloid-derived suppressor cells and reduce cancer cell growth (Li et al., 2016; Kim et al., 2017; Prima et al., 2017; Goyal et al., 2018). An improved understanding of the relationship between fatty acid metabolism and the immune profile of the TMEs can help find other therapeutic targets and extend the clinical benefit of immunotherapy to more patients. In this study, the difference in the immune profile of LUAD was compared between the high-risk and low-risk score patients. Although there was no difference in the expression level of PD1 and CTLA4, the high-risk score cohort showed higher PDL1 expression, indicating that the high-risk score patients can potentially benefit from anti-PD1/PDL1 antibodies.

There are some limitations to be noted in the study for the reference of future studies. First, selection bias potentially exists for the inevitable retrospective nature, and the genomic and clinical data are extracted from the public database. Second, the sample size of this study is small, and further studies are warranted to validate the results.

## Conclusion

In summary, we identified 38 fatty acid metabolism-related gene-based FARS which could accurately predict the survival outcome of LUAD patients. Patients with higher FARS can potentially benefit from anti-PD1/PDL1 immunotherapy. In addition, the mechanisms of the genes in the FARS affecting prognosis are worthy of further research to develop new gene-targeted drugs.

## Data Availability

The datasets presented in this study can be found in online repositories. The names of the repository/repositories and accession number(s) can be found in the article/Supplementary Material.

## Abbreviations

AJCC TNM, American Joint Committee on Cancer Tumor Node Metastasis; AUC, area under the curve; FARS, fatty acid-related risk score; CTLA4, cytotoxic T-lymphocyte-associated protein 4; GEO, Gene Expression Omnibus; GOBP, gene ontology biological process; LASSO, least absolute shrinkage and selection operator; LUAD, lung adenocarcinoma; NSCLC, non-small cell lung cancer; OS, overall survival; PD1, programmed cell death protein 1; PDL1, programmed death-ligand 1; pStage, pathological stage; ssGSEA, single-sample Gene Set Enrichment Analysis; TCGA, The Cancer Genome Atlas; TMEs, tumor microenvironments; tROC, time-dependent receiver operating characteristic curves; WGCNA, Weighted Gene Co-expression Network Analysis.
