# Supplementary material for: Characterization of Fatty Acid Metabolism in Lung Adenocarcinoma
Source: Front Genet. 2022 Jul 14;13:905508. doi: 10.3389/fgene.2022.905508 (PMC9329533; doi:10.3389/fgene.2022.905508)
Supplement: Supplementary file 1 [file DataSheet1.zip › Supplementary material 2.docx]

**TABLE 1** Baseline characteristics of LUAD patients

| Variables | TCGA | GSE72094 | GSE31210 | P |
| --- | --- | --- | --- | --- |
|  | n=468 | n=393 | n=226 |  |
| Age | 66.0 (59.0-72.0) | 70.0 (64.0-77.0) | 61.0 (55.0-65.0) | <0.001 |
| Sex |  |  |  | 0.851 |
| Female | 254 (54.3%) | 219 (55.7%) | 121 (53.5%) |  |
| Male | 214 (45.7%) | 174 (44.3%) | 105 (46.5%) |  |
| pStage |  |  |  | <0.001 |
| I | 256 (54.7%) | 254 (64.6%) | 168 (74.3%) |  |
| II | 113 (24.1%) | 67 (17.0%) | 58 (25.7%) |  |
| III | 75 (16.0%) | 57 (14.5%) | 0 (0.0%) |  |
| IV | 24 (5.1%) | 15 (3.8%) | 0 (0.0%) |  |

Continuous variables are presented as median (interquartile range) and compared using Kruskal Wallis test, and categorical variables are presented with number (percentage) and compared using Chi-square test. LUAD, lung adenocarcinoma; pStage, pathological stage.
